# Annotations

**Published:** 1893-10-28

**Authors:** 


### ANNOTATIONS.

### Sir Andrew Clark's Illness.

The medical profession is not by nature demonstrative, but the sudden, unexpected, and alarming seizure of Sir Andrew Clark on Thursday week, whilst in the pursuit of his ordinary duties, deeply touched many a heart which is not accustomed to yield to undue emotion. Sir Andrew Clark has played a conspicuous part in medicine, and indirectly in public life for a good many years. His own sword carved out for him the way to eminence. He bears the scars of many a conflict—conflicts not the less real because waged in the silence and secrecy of his own heart. But patience, courage, and an unyielding determination have had their reward, not only in the attainment of distinction, but of distinction with general approval, and in many cases with personal affection. Sir Andrew Clark is a kind man, and in the best sense a good man. He can put himself in the place of other men, and look at things from their standpoint. He can sympathise with the unsuccessful and the half-successful. He is therefore, to many people, something of a hero, a man beloved. The sympathy of the medical profession with him is profound and universal. That the public shares in this sympathy is evidenced by the fact that last Saturday and Sunday there were no fewer than seven hundred callers at his house in Cavendish Square. Her Majesty the Queen and the Prince and Princess of Wales share in the universal sympathy. As showing how thoroughly Sir Andrew Clark has deserved the regard of his own profession a little incident of the day before his seizure may be mentioned. He was to attend a meeting of the Royal College of Physicians. By some untoward circumstance he was half an hour late. He was deeply distressed by the accident, and declared that in a period of sixteen years in which he had attended the meetings of the Fellows he had never been late before. We, among thousands of others, are grateful to hear of Sir Andrew's steady progress towards recovery, It is pleasant to know that he is under the nursing care of one of the nurses who worked in his old wards at the London Hospital. Dr. Russell Reynolds, Dr. Stephen Mackenzie, and Dr. Barlow are in attendance, and under their care, we earnestly hope that Sir Andrew will be completely restored to health, and to the active duties of the profession of which he is so distinguished an ornament.

### Cruelty in Irish Parents.

The case of Daniel Phelan and his wife, who were convicted at the Chester Quarter Sessions the other day of savage and continuous cruelty to their two infant children, reminds us of the case of Mrs. Montague. The Phelans are Irish, and Mrs. Montague was Irish; the Phelans are, presumably, Roman Catholics, as Mrs. Montague was a Roman Catholic; the Phelans are persons of means and education, as Mrs. Montague was a lady of position and substance. We need not recount the acts of cruelty in either case; the facts must be fresh in the minds of all newspaper readers. But that any educated mother should treat her infant of less than two years as Mrs. Phelan treated hers, would be incredible if it had not been proved to be undeniably true. There are savages among the ignorant in all countries, but for children to be thus brought up by educated and presumably religious people, is so shocking and heart-sickening that one is inevitably compelled to ask oneself, How can such things be? Are the Phelans sane? And if they are sane, what is the possible origin of their stupid savagery? Is it their Irish blood? If it be, the Society for the Prevention of Cruelty to Children must increase its agents a hundredfold in Ireland. But if it be not their Irish blood, is it their Romish religion? If so, the teachers of the Roman Catholic Church must look to the doctrines they enunciate, and to the intellectual condition of their flocks. We do not believe that it is either the Irish blood or the Romish religion. But what, then, is it? Such barbarity as that of the Phelans and the Montagues is a blot upon civilisation, and every one of us is bound to try to discover the origin of it, with a view to the restriction of similar savagery within the narrowest possible limits. Ireland and her religion should authoritatively disclaim any participation in horrors of this kind.

### Object Lessons from Lanarkshire.

"An ounce of fact," we are often told, "is worth a pound of theory." Sir William Hamilton would not have admitted the universal truth of the statement, nor do we. But it is a useful statement all the same especially for what are called "common-sense" minds. Dr. McLintock, the Medical Officer of Health for Lanarkshire, whom we have learnt to consider a man of competence and authority, gives, in his annual report for 1892, some instances of typhoid outbreaks which are very convincing in their demonstration of the dangerousness of contaminated water. In the towns of Blantyre and Cambuslang there were epidemics of typhoid fever of marked severity in 1892. The maximum severity of the epidemic in the former town was in a district known by the name of Clyde Rows, and in the latter in a locality known as Spittal. Dr. McLintock made special investigations in both cases. At Clyde Rows there were 18 houses, and in these 18 houses 23 persons were attacked with typhoid fever. The inhabitants of the houses obtained their water supply from a watercourse where two small streams met, one of the streams passing through a drain "in close proximity to the whole of the privies and ashpits behind the houses." The result we have already considered. One naturally asks whether those people who lived in Clyde Rows were Scots or savages? Had they shared in the advantages of the Scottish system of elementary education of which we hear so much? No doubt they had, but it is evident that they had put their education to a very poor use. Why, one thinks, should they not have protected themselves and their families by declining to drink water from so vile and polluted a source. Clearly the common public has yet much to learn; and there must be no relaxation of vigilance on the part of medical officers of health and other sanitary authorities. The outbreak at Spittal was almost more convincing in its demonstration of the dangers of polluted water than that at Clyde Rows. "By the breaking down of certain machinery the water supply of Spittal failed, and the inhabitants had recourse to a neighbouring pit, employing about 500 men. Some of the people drank the pit water, whilst others did not. Of those who had typhoid fever the majority, or nearly all, had drunk the pit water. Among those who had had their water supply from unpolluted sources there was no outbreak of typhoid at all. We do not need to point the moral in these cases. It is for public authorities to recognise their full significance, and equally for grown up men and women who are responsible for their own health and that of their families and neighbours to recognise that significance. Sanitary knowledge and sanitary faith are to be promulgated diligently among all ranks of the people. Such promulgation will in due time be followed by a sanitary millennium, at any rate, so far as freedom from typhoid, cholera, or some other like diseases are concerned. No doubt that millennium is yet a long way off, but the duty of medical officers and all who have sanitary knowledge is manifestly to keep "pegging away."

